# The Association between Acute Myocardial Infarction-Related Outcomes and the Ramadan Period: A Retrospective Population-Based Study

**DOI:** 10.3390/jcm11175145

**Published:** 2022-08-31

**Authors:** Batya Betesh-Abay, Arthur Shiyovich, Shani Davidian, Harel Gilutz, Walid Shalata, Ygal Plakht

**Affiliations:** 1 Department of Nursing, Recanati School for Community Health Professions, Faculty of Health Sciences, Ben-Gurion University of the Negev, Beer-Sheva 8410501, Israel; 2 Department of Cardiology, Rabin Medical Center, Petah Tikva, Israel and Sackler Faculty of Medicine, Tel Aviv University, Tel Aviv 4941492, Israel; 3 Department of Public Health, Faculty of Health Sciences, Ben-Gurion University of the Negev, Beer-Sheva 8410501, Israel; 4 Department of Emergency Medicine, Soroka University Medical Center, Beer-Sheva 8457108, Israel; 5 Goldman Medical School, Faculty of Health Sciences, Ben-Gurion University of the Negev, Beer-Sheva 8410501, Israel; 6 The Legacy Heritage Oncology Center, Dr. Larry Norton Institute, Soroka University Medical Center, Beer-Sheva 8457108, Israel

**Keywords:** Muslims, Ramadan, acute myocardial infarction, incidence, all-cause mortality, population-based study

## Abstract

Fasting throughout the Muslim month of Ramadan may impact cardiovascular health. This study examines the association between the Ramadan period and acute myocardial infarction (AMI)-related outcomes among a Muslim population. The data were retrospectively extracted from a tertiary hospital (Beer-Sheva, Israel) database from 2002–2017, evaluating Muslim patients who endured AMI. The study periods for each year were: one month preceding Ramadan (reference period (RP)), the month of Ramadan, and two months thereafter (1840 days in total). A comparison of adjusted incidence rates between the study periods was performed using generalized linear models; one-month post-AMI mortality data were compared using a generalized estimating equation. Out of 5848 AMI hospitalizations, 877 of the patients were Muslims. No difference in AMI incidence between the Ramadan and RP was found (*p* = 0.893). However, in the one-month post-Ramadan period, AMI incidence demonstrably increased (AdjIRR = 3.068, *p* = 0.018) compared to the RP. Additionally, the highest risk of mortality was observed among the patients that underwent AMI in the one-month post-Ramadan period (AdjOR = 1.977, *p* = 0.004) compared to the RP. The subgroup analyses found Ramadan to differentially correlate with AMI mortality with respect to smoking, age, sex, diabetes mellitus, and hypertension, suggesting the Ramadan period is a risk factor for adverse AMI-related outcomes among select Muslim patients.

## 1. Introduction

Acute myocardial infarction (AMI) is rigorously explored in medical research given the pervasive prevalence and incidence of AMI events, and the interconnected strains incurred on patients, their families, and the public health system as a result [1,2]. Several environmental and behavioral factors have been investigated as AMI predictors, including: workplace conditions, socioeconomic status, changes in daylight savings time, cigarette smoking status, eating, sleeping habits, and more [3,4,5,6,7].

The Muslim month of Ramadan instructs healthy adults to abstain from food consumption, as well as the intake of medications, during daytime hours [8]. The religious mandate entails a profound shift in eating and sleeping patterns, with two customary night-time feasts replacing conventional daily meals [9]. Observance of Ramadan temporarily alters overall calorie intake and dietary routine, medication regimens, and sleep habits, among other accompanying behavioral and social changes pertinent to the time [9,10]. Studies examining Ramadan and cardiovascular morbidity mainly indicate unchanged or even advantageously associated outcomes [11,12,13,14]. A few works, however, suggest that the observance of Ramadan can potentiate adverse health manifestations among select patient groups, including increased risk of cardiovascular events [15], deterioration in renal function [16], and disruption in glucose homeostasis [17].

In the southern district of Israel, the demographic population (~770,000 residents in 2019) is predominantly composed of Muslim (~35%) and Jewish inhabitants (~65%) [18,19]. The large-scale regional hospital singly serves these coexistent ethnic/religious groups. Given this unique setup, we speculated about designing a population-based study to evaluate Ramadan with respect to cardiovascular health. Furthermore, we hypothesized expanding the scope of our investigation to assess the pre- and post-Ramadan periods of transitions out of and into habitual lifestyle. The primary aim of this study was to examine the association between the month of Ramadan and the surrounding periods (one month prior and two months following) and AMI-related health outcomes among the Muslim and non-Muslim local populations.

## 2. Materials and Methods

### 2.1. Study Population and Setting

This retrospective population-based study included patients admitted to Soroka University Medical Center (SUMC) with a diagnosis of AMI from 1 January 2002 to 25 October 2017. SUMC is a tertiary referral center (~1200 beds), serving the metropolitan area of Beer-Sheva and the southern Israel region. All participants were 18 years or older and residents of the southern region of Israel. Patients with non-Israeli citizenship or unclassified nationality were excluded.

### 2.2. Study Groups and Time Periods

The Muslim study sample was the focal group of this research. The non-Muslim population served as a convenience reference group, with the Ramadan fast as the exposed variable among the Muslim group only. Time periods were: (i) the month of Ramadan (spanning 28 or 29 days, depending on the year) in its respective time each year (based on a lunar calendar: Ramadan occurs at different annual dates each Gregorian calendar year [8]), (ii) one month prior to Ramadan, (iii) one month following Ramadan, and iv) two months following Ramadan (see Supplementary Table S1).

### 2.3. Data Collection and Variables

Medical and demographic data were extracted from the SUMC electronic files for all hospitalizations of patients during the study timeframe, as previously described in the Soroka Acute Myocardial Infarction (SAMI) project [20,21]. Using the International Classification of Diseases, Ninth Revision, Clinical Modification (ICD-9-CM) discharge codes, AMI diagnoses were determined as follows: ST-elevation MI (STEMI): 410.0*–410.6* and non-ST elevation MI (NSTEMI): 410.7*–410.9*. Anemia referred to low blood hemoglobin levels. Diabetes mellitus diagnosis was applied when hemoglobin A1C levels ≥ 6.5%. Patients were defined as having renal failure if they were either on hemodialysis or with an estimated glomerular filtration rate (eGFR) < 60 mL/min/1.73 m^2^. A diagnosis of dyslipidemia was given when low-density lipoprotein levels were ≥100 mg/dL or by relevant ICD-9-CM codes. Severe left ventricular dysfunction was defined as left ventricular ejection fraction < 30%. Additional morbidities were also determined via ICD-9-CM. Further extracted information included AMI management, as well as laboratory, angiographic, and echocardiographic findings. Characteristics of hospitalization were documented including admission information and length of stay (LOS) (measured in days). Survival status and the date of death for each patient were obtained from the Ministry of Interior population registry. The study protocol conformed to the ethics guidelines of the 1975 Declaration of Helsinki as reflected in a priori approval by the human research committee of the institution. 

In addition, data on the general population was referenced from the Israel Central Bureau of Statistics database [18] for each corresponding study year for the south of Israel, stratified by population size, sex, age, and nationality. 

### 2.4. Outcomes

For each study period, two outcomes were defined: (1) incidence of AMI, calculated as the number of AMI admissions in the referenced general Muslim and non-Muslim populations; and (2) one-month post-AMI all-cause mortality was assessed using the individual data of patients.

### 2.5. Statistical Analysis

The data analysis was performed using IBM SPSS Statistics 26 software. Patient characteristics are presented as mean and standard deviation (SD) for continuous variables and as *n* and percent for categorical data. A comparison of the characteristics of patients between the study periods was performed using the analysis of variance (ANOVA) and chi-square tests.

#### 2.5.1. Incidence

The data are presented separately for the Muslim and non-Muslim groups for each study period as the average annual number of hospital admissions, and unadjusted and adjusted annual AMI incidence rates (AdjIRs). Unadjusted IR was calculated for each study year as the number of AMI admissions divided by the size of the adult population (aged 20 years and over) in the geographic region of the study (Beer-Sheva district, southern Israel) in a given year. AdjIRs were calculated using direct standardization for age and sex, in which the standard population was the Israeli population in the year 2017. These data are presented as mean and SD. We used the ANOVA tests with Bonferroni post hoc tests for a comparison of incidence data between the study periods in a univariate level. In addition, comparison of AdjIRs between the study time periods was performed using generalized linear models (GLM), scaled by population size per pertaining study year. The results of the models are presented as incidence rate ratios (IRRs) and 95% confidence intervals (CIs) with the reference period as one month before Ramadan.

#### 2.5.2. Mortality

Mortality data are presented for Muslim and non-Muslim groups as *n* and percent, and a comparison between the study time periods was performed using chi-square test. In addition, average annual mortality rates were calculated and compared using ANOVA test. Generalized estimating equation (GEE) models were built, in univariate and multivariate levels, considering recurrent AMI admissions of patients over the study years. These models included the parameter of study period and the baseline characteristics of the patients, statistically associated with the outcome. The results of analytical models are presented as odds ratios (ORs) and adjusted ORs (AdjORs) with 95% CIs. 

#### 2.5.3. Subgroup Analysis

Subgroup analysis (multivariate GEE models) was performed among the Muslim population. The models were built stratifying the file by sex, age, and main cardiovascular risk factors.

For each test, *p*-values of <0.05 (two-tailed) were considered statistically significant.

## 3. Results

### 3.1. Incidence

The data for a total of 1840 days were collected, comprising 460 days of Ramadan and the remaining days making up the surrounding one month prior and two months thereafter, for each respective year of this study (2002–2017). During this period, 5848 AMI hospitalizations were documented. Among them, 877 were Muslim patients, and 4954 were non-Muslim patients.

Among the Muslim study group, the annual average number of AMI hospitalizations across all defined study periods was 13.70 (SD = 4.74), the annual mean incidence (/1000 persons) was 2.04 (SD = 0.70), and the annual AdjIR was 5.49 (SD = 2.83). Among the non-Muslim population, the annual average number of AMI hospitalizations was 77.67 (SD = 14.95), the annual mean incidence was 3.03 (SD = 0.70), and the annual AdjIR was 2.77 (SD = 0.77).

**Figure 1:**
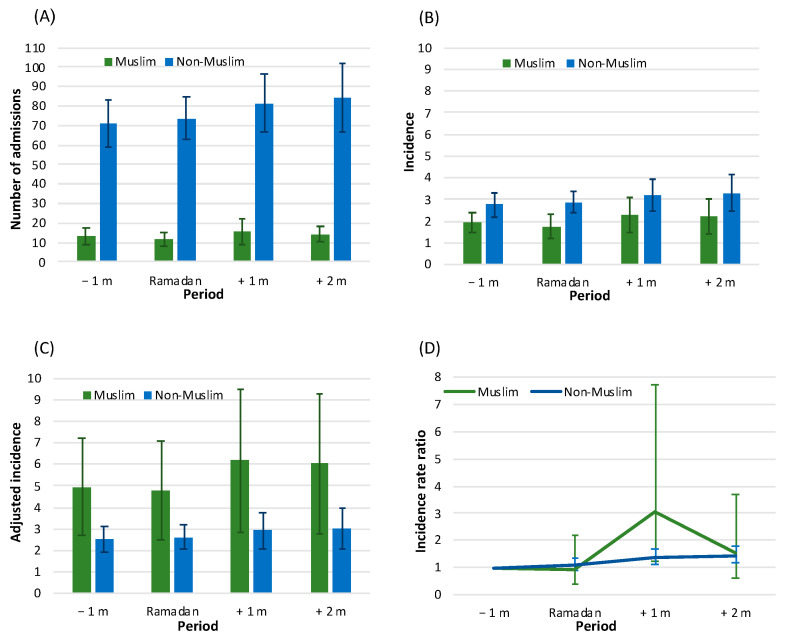
Incidence data of acute myocardial infarction (AMI) by study time period among Muslims and non-Muslims: (**A**) number of admissions, (**B**) unadjusted incidence (/1000), (**C**) adjusted incidence (/1000), and (**D**) incidence rate ratio. Number of admissions and incidence rates (/1000) (panels **A**–**C**) are presented as annual average and standard deviation (SD); (Panel **C**)—direct age and sex adjustment, standard population—the Israeli population in the year 2017; (Panel **D**)—incidence rate ratio (IRR) and 95% confidence interval (CI) from generalized linear models (GLM), scaling by the population size in each study year. Legend: −1 m—month prior to Ramadan, Ramadan—month of Ramadan, +1 m—one-month post-Ramadan, +2 m—two-months post-Ramadan.

Figure 1 depicts the incidence data of AMI events by study period among Muslim and non-Muslim populations. Regarding the Muslim population, the results of univariate comparisons demonstrate significant differences between the Ramadan and one-month post-Ramadan periods in the annual number of AMI admissions: 11.69 (SD = 3.48) vs. 15.63 (SD = 6.59), *p* = 0.019; and in annual incidence rates: 1.74 (SD = 0.55) vs. 2.27 (SD = 0.82), *p* = 0.032. No statistically significant differences in AdjIRs between the periods were found. The results of the GLM model show that in the one-month post-Ramadan period, AMI incidence demonstrably increases with an AdjIRR of 3.068 (95% CI: 1.217–7.734, *p* = 0.018) in comparison to the one month prior, while no statistical significance was found throughout the remaining time periods. Thus, for the period of Ramadan, the findings are as follows: AdjIRR = 0.945, 95% CI: 0.412–2.166, *p* = 0.893; and for the period of two months after Ramadan: AdjIRR = 1.510, 95% CI: 0.615–3.708, *p* = 0.368.

Contrastingly, among the non-Muslim population, no difference was found in the incidence between the period before Ramadan and the month of Ramadan itself: AdjIRR = 1.085, 95% CI: 0.895–1.317, *p* = 0.407. For the periods after Ramadan, a slight statistically significant increase was noted compared to the period before Ramadan. The findings observed are: for the month after Ramadan, AdjIRR = 1.359, 95% CI: 1.111–1.662, *p* = 0.003; and for two months after: AdjIRR = 1.435, 95% CI: 1.170–1.760, *p* = 0.001.

### 3.2. Baseline Data by Time Period

The demographic and clinical baseline data of the Muslim study cohort, tabulated by study time periods (one month preceding Ramadan, the month of Ramadan, and two months thereafter) are presented in Table 1. Among this population, the average age (for all time periods) was 60.81 (SD = 14.30) years, 72.6% were males, nearly half of the AMI events were STEMI (46.2%), and roughly 75% underwent invasive intervention (percutaneous coronary intervention (PCI) or coronary artery bypass graft (CABG)). The most frequent comorbidities among the Muslim population were: chronic ischemic heart disease (CIHD), diabetes mellitus, dyslipidemia, hypertension, and smoking.

Among the Muslim group, patients who underwent AMI during Ramadan tended to be older in comparison to the other study time periods. For the majority of the analyzed parameters, no statistically significant difference was found between study periods. The demographic and clinical baseline data of the non-Muslim study population are presented in Supplementary Table S2.

### 3.3. Mortality by Time Period

**Figure 2:**
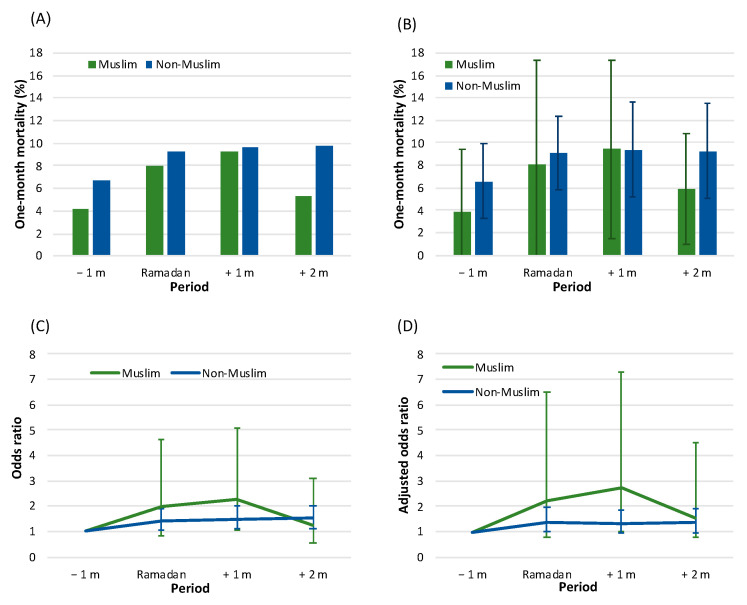
One-month mortality by study time period among Muslims and non-Muslims: (**A**) mortality rates (%), (**B**) annual mortality rates (mean and standard deviation), (**C**) unadjusted relative risk for mortality (odds ratio and 95% confidence interval (CI)), and (**D**) adjusted relative risk for mortality (adjusted odds ratio and 95% CI). Based on the results of the multivariate models (separately for Muslims and non-Muslims)—see Supplementary Table S3A,B. Legend: −1 m—month prior to Ramadan, Ramadan—month of Ramadan, +1 m—one-month post-Ramadan, +2 m—two-months post-Ramadan.

Of the 877 hospitalizations among the Muslim population, there were 59 (6.7%) recorded deaths (an annual average mortality rate of 6.85%, SD = 7.32). The lowest mortality rate was found in the period of one month before Ramadan (9/213, 4.2%), and the highest death rate was noted one month following the cessation of Ramadan (23/250, 9.2%; see Figure 2A). However, no statistically significant differences in mortality rates were found across the study time periods (*p* = 0.121). In addition, no significant difference in the mean annual mortality rate between the periods was found (*p* = 0.142, see Figure 2B).

With regard to the non-Muslim study population, there were 439 (8.9%) deaths out of the 4954 hospitalizations (an annual average mortality rate of 8.59%, SD = 3.87). There was a significant difference in the monthly mortality rate between the defined time periods (*p* = 0.030), with a gradual increase from 76/1137 (6.7%) in the period of one month before Ramadan up to 130/1333 (9.8%) in the period of two-month post-Ramadan (Figure 2A). No significant difference in the mean annual mortality rate between the periods was found (*p* = 0.115, see Figure 2B).

Figure 2C depicts results of the univariate regression models (OR values) of one-month mortality. Among the Muslim population, the time period with the highest risk of mortality occurs one month following the month of Ramadan in comparison to the month preceding Ramadan (OR = 1.977, 95% CI: 1.039–5.078, *p* = 0.040). Among the remaining time periods (including the month of Ramadan itself), no statistical significance was present. In the non-Muslim population, the time periods with the highest risk of mortality are during Ramadan and the successive month, compared to the previous period where no statistical significance was found. (OR values were ~1.4–1.5 with statistical significance relative to the time period preceding Ramadan).

The results of the multivariate regression mortality model are displayed in Figure 2D. Following adjustment for potential confounders, among the Muslim population, a significantly increased mortality risk was found in the one-month period subsequent to Ramadan in juxtaposition to the month prior to Ramadan (AdjOR = 2.709, 95% CI: 1.008–7.280, *p* = 0.048). For the non-Muslim population, no association was noted between the study time periods and risk of mortality. AdjOR values for each post-Ramadan period were ~1.3–1.4, with borderline statistical significance.

The results of the multivariate models for one-month mortality among Muslim and non-Muslim population are recorded in the Supplementary Table S3A,B.

### 3.4. Subgroup Analysis

**Figure 3:**
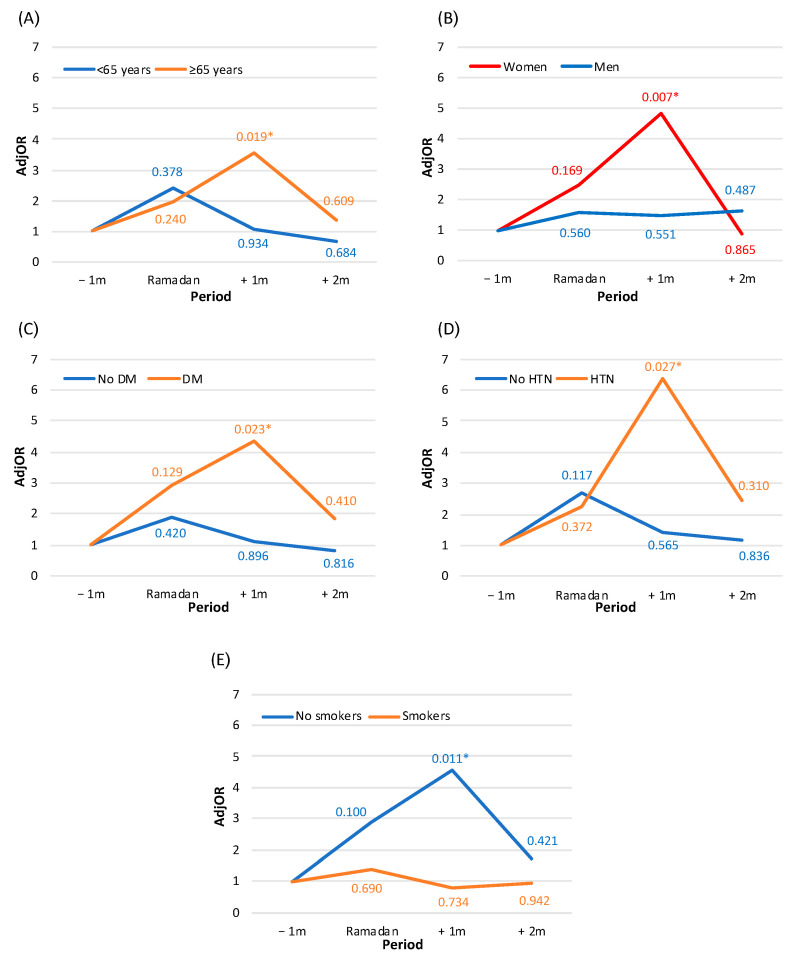
Relative risk for one-month mortality by study period among Muslims: subgroup analysis by (**A**) age, (**B**) sex, (**C**) diabetes mellitus (DM), (**D**) hypertension (HTN), and (**E**) smoking status. The figure presents the results of the multivariate models (separate model for each panel). Each model is adjusted for: year of the event, age, type of acute myocardial infarction, left ventricular dysfunction, chronic ischemic heart disease, renal diseases, anemia, and gastrointestinal bleeding. The numbers present *p*-values of adjusted odds ratios (AdjOR) compared to the period of one-month prior to Ramadan. *—statistically significant interaction (*p*-for-interaction < 0.05). Legend: −1 m—month prior to Ramadan, Ramadan—month of Ramadan, +1 m—one-month post-Ramadan, +2 m—two-months post-Ramadan.

The results of subgroup analysis show that, compared to the one-month period before Ramadan, there was an increased risk of mortality in the one-month post-Ramadan period among the following patient groups: the elderly (aged 65 and over) (AdjOR = 3.539, 95%CI: 1.226–10.215, *p* = 0.019); women (AdjOR = 4.839, 95%CI: 1.530–15.303, *p* = 0.007); patients with diabetes mellitus (AdjOR = 4.345, 95%CI: 1.220–15.471, *p* = 0.023); patients with hypertension (AdjOR = 6.376, 95%CI: 1.230–33.044, *p* = 0.027); and nonsmokers (AdjOR = 4.543, 95%CI: 1.410–14.639, *p* = 0.011) (Figure 3). Among these groups, no difference was found between the periods before Ramadan and the remaining study periods. However, the time period of one month after Ramadan was notably prominent. No difference in the relative risk of mortality was apparent according to the division of further comorbidities (dyslipidemia, kidney diseases, anemia, etc.).

## 4. Discussion

This study examined the Muslim month of Ramadan and AMI-related outcomes, encompassing the time period of one month before the commencement of Ramadan and extending until two months after Ramadan ended, over a period of 16 years. Our findings show that among the Muslim study population, AMI incidence during Ramadan was not different from that in the month before Ramadan. However, the highest risk of AMI incidence was one month after the completion of Ramadan. With regard to mortality, an increase was observed during Ramadan compared to the period before Ramadan (AdjOR ~ 2.2). In addition, the highest risk of mortality was found in the month after Ramadan (AdjOR ~ 2.7). Nonetheless, given the relatively small sample size, trended mortality dynamics throughout the days of Ramadan proper were not analyzed. It is possible that most of the deaths were towards the end of the Ramadan month.

While the current study did not have exact information with regards to patient fasting throughout Ramadan, it is important to emphasize that observance of the Ramadan fast by the local Muslim population is considered culturally mainstream and a known matter in hospital settings. The Pew Research Center reliably reported trends of 83% of Muslims in Israel fasting throughout Ramadan in 2016 [22]. Thus, we discretionarily and reasonably concluded that an ample number of the Muslim cohort in the current study abided by the fast, in turn, deeming a solid rationale for the conduction of the current study.

Our findings seem to somewhat differ from certain reporting in literature on Ramadan and AMI-related outcomes. In a systematic review and meta-analysis examining Ramadan and cardiovascular health, findings showed no significant difference in AMI- related outcomes among fasting patients compared to those who did not fast [13]. Furthermore, beneficial cardiometabolic outcomes were reported in correlation with Ramadan, including reduction in systolic blood pressure, improved cholesterol profiles, and a decreased rate of cerebral infarct, among others [23,24]. The discussions surrounding these results generally attribute benefits to reduced caloric intake [25] and refrainment from cigarette smoking [26]. These parameters were not assessed in the current study due to the dearth of data availability. It is feasible that exposure to these risk factors is varied among our local population in contrast to the study samples examined in previous works. Notwithstanding, the body of research on Ramadan and cardiovascular health is still deemed inconclusive [13,27], as an additional study prospectively examining the effects of Ramadan fasting on renal functions among diabetic patients found a notable decline in eGFR at six-weeks post-Ramadan [28]. NasrAllah et al. [15] followed 131 patients with stabilized chronic kidney disease who voluntarily fasted during the month of Ramadan. Their findings showed fasting to correlate with a marked rise in serum creatinine and adverse cardiovascular events. In this context, we were impelled to perform a subgroup analysis among the Muslim study group for cardiovascular risk factors; this stratification indicated prevalence of select parameters to serve as predictive factors for AMI-related adverse outcomes during the defined study periods. We found that AMI mortality more severely associated with Ramadan with respect to select patient subgroups including: nonsmokers, those above 65 years in age, females, patients with diabetes mellitus, and patients with hypertension. It was interesting that specifically nonsmokers displayed increased AMI mortality under the study design. In this regard, we can speculate that perhaps this relates to sex-related differences in smoking statuses, with the established finding that the female population smokes less than males [29].

Patient assessment, education, and monitoring surrounding the Ramadan period is critical. A study implementing an active interventional pre-Ramadan patient education and monitoring program found that Muslims with diabetes who fasted during Ramadan exhibited optimal self-managing by means of effective tele-monitoring support and intervention. Patients who utilized this program also showed lessened complications during Ramadan in contrast to pre-Ramadan with regard to glycemic control and metabolic profiling [30].

In the current study, the adverse association between Ramadan and AMI-incidence was not noted during the month of Ramadan itself but rather transpired in the month succeeding. Some research that examined similar parameters and extended their study timeframe to the one month after Ramadan did not find an increase in AMI incidence [31,32]. We hypothesize that this lag in the current study is multifactorial and may derive from patterns of transitioning out of the extended daily fasting and resuming habitual lifestyle, including dietary and sleeping regimens, and social and behavioral aspects. Additional aspects likely include local living conditions and socioeconomic considerations. Many of the local Muslim population in the current study live a seminomadic lifestyle in rural housing, and disparities in utilization and access to healthcare services among this population have been reported [33].

Potential pathophysiological mechanisms linking the Ramadan fast and AMI events may pertain to underlying chronic illness, particularly diabetes mellitus, as the literature shows that diabetic patients who fast for the Ramadan month are at risk of acute cardiometabolic complications due to unbalanced glycemic control and increased incidence of diabetic ketoacidosis [34,35,36]. As seen in the subgroup analysis of the current study, specifically patients with diabetes showed increased incidence in adverse cardiovascular outcomes. In addition, the Ramadan nocturnal feasts may cause extended disrupted circadian rhythm, which has been shown to intrinsically correlate with cardiovascular health, due to observed alterations in endothelial activity and distorted platelet and thrombotic function [37]. An additional rationalizing mechanism may be due to dysregulated immunity [38].

Moreover, local seasonal factors possibly contribute to our findings; the months of Ramadan examined in the current study primarily spanned across months that are of a particularly hot and dry climate in the desert setting of our study. In this context, we emphasize that, although less dramatic, differences of AMI-incidence were also found among the non-Muslim study group during this time. Previous reports have linked biometeorological aspects and MI, finding that extreme weather conditions tend to associate with cardiovascular morbidity and mortality [39,40].

The current study model analyzed two side-by-side populations living in the same climate. Therefore, examining morbidity during the study periods may seemingly neutralize the influence of environmental factors such as weather. However, it is feasible that the non-Muslim population differs from the Muslim population in further parameters pertaining to lifestyle, culture, healthcare accessibility, and more, extending beyond just the Ramadan fast. Another element to mention was the factor of age. While the non-Muslim regional population is generally older, the Muslim cohort who endured AMI in the current study were on average 8 years younger than the non-Muslims. Therefore, when examining raw incidence rates, we found lower rates among the Muslim study group; however, after adjustment per age, the rates were markedly higher among the Muslim group.

### Limitations

This was a single-center retrospective design that did not perform direct analytical comparison between the two study populations. Regarding study groups, patients did not report if they observe Ramadan fasting or not. No further information regarding diet, medication intake, sleeping, or workplace habits were available for assessment. Moreover, data regarding pharmacotherapy throughout the hospitalization and follow-up of patients, in particular with respect to adherence to medical guidelines, were missing. Additional limitations were: patients who experienced fatal AMI events and died prior to hospitalization were not included in the analysis; local patients who received treatment at alternative hospitals were also not included; the cause of patient deaths among our cohorts was not known as it may not have been due to a cardiac event; data regarding nonfatal cardiovascular-related outcomes (such as major adverse cardiac and cerebrovascular events, MACCE) during the follow-up period were also unavailable for analysis; and we did not monitor patients to see if there were any changes in physiological indices during the month of Ramadan and the surrounding time periods.

## 5. Conclusions

This study investigated Ramadan and AMI-related outcomes in a population-based study model, observing that the post-Ramadan period serves as a risk factor for adverse AMI-related outcomes among the Muslim population. Effective patient education programs are warranted. Further evaluation of parameters is necessary in a multicenter prospective study design.

## Figures and Tables

**Table 1:** Baseline Characteristics of the Muslim Study Population by Period.

	−1 m	Ramadan	+1 m	+2 m	Total	*p*
*N*	213	187	250	227	877	
**Demographics**						
Age (years), Mean (SD)	58.48 (14.64)	62.10 (14.91)	60.79 (13.82)	61.96 (13.79)	60.81 (14.30)	0.034
65–75	35 (16.4)	42 (22.5)	57 (22.8)	62 (27.3)	196 (22.3)	0.064
≥75	31 (14.6)	35 (18.7)	35 (14.0)	40 (17.6)	141 (16.1)	
Sex, Males	158 (74.2)	135 (72.2)	187 (74.8)	157 (69.2)	637 (72.6)	0.524
**Cardiac diseases**						
Cardiomegaly	21 (9.9)	25 (13.4)	30 (12.0)	37 (16.3)	113 (12.9)	0.230
Supraventricular arrhythmias	23 (10.8)	29 (15.5)	33 (13.2)	31 (13.7)	116 (13.2)	0.577
CHF	36 (16.9)	38 (20.3)	53 (21.2)	49 (21.6)	176 (20.1)	0.601
Pulmonary heart disease	14 (6.6)	24 (12.8)	20 (8.0)	30 (13.2)	88 (10.0)	0.043
CIHD	184 (86.4)	159 (85.0)	217 (86.8)	195 (85.9)	755 (86.1)	0.959
**Cardiovascular risk factors**						
Renal diseases	18 (8.5)	22 (11.8)	26 (10.4)	25 (11.0)	91 (10.4)	0.721
Diabetes mellitus	98 (46.0)	100 (53.5)	124 (49.6)	122 (53.7)	444 (50.6)	0.332
Dyslipidemia	154 (72.3)	133 (71.1)	175 (70.0)	166 (73.1)	628 (71.6)	0.885
Hypertension	99 (46.5)	100 (53.5)	120 (48.0)	118 (52.0)	437 (49.8)	0.439
Obesity	45 (21.1)	44 (23.5)	39 (15.6)	51 (22.5)	179 (20.4)	0.149
Smoking	139 (65.3)	113 (60.4)	165 (66.0)	124 (54.6)	541 (61.7)	0.045
PVD	16 (7.5)	18 (9.6)	20 (8.0)	12 (5.3)	66 (7.5)	0.407
Family history of IHD	19 (8.9)	12 (6.4)	23 (9.2)	18 (7.9)	72 (8.2)	0.732
**Other disorders**						
COPD	35 (16.4)	38 (20.3)	48 (19.2)	53 (23.3)	174 (19.8)	0.333
Neurological disorders	23 (10.8)	24 (12.8)	29 (11.6)	27 (11.9)	103 (11.7)	0.939
Malignancy	5 (2.3)	3 (1.6)	5 (2.0)	5 (2.2)	18 (2.1)	0.959
Anemia	70 (32.9)	67 (35.8)	87 (34.8)	93 (41.0)	317 (36.1)	0.323
GI bleeding	4 (1.9)	4 (2.1)	3 (1.2)	4 (1.8)	15 (1.7)	0.889
Schizophrenia/psychosis	4 (1.9)	3 (1.6)	3 (1.2)	3 (1.3)	13 (1.5)	0.935
Alcohol/drug addiction	2 (0.9)	0 (0)	2 (0.8)	4 (1.8)	8 (0.9)	0.312
History of malignancy	0 (0)	5 (2.7)	1 (0.4)	4 (1.8)	10 (1.1)	0.039
**Administrative characteristics of the hospitalization**						
LOS, >7 days	85 (39.9)	83 (44.4)	102 (40.8)	75 (33.0)	345 (39.3)	0.111
STEMI	106 (49.8)	76 (40.6)	123 (49.2)	100 (44.1)	405 (46.2)	0.192
**Results of echocardiography**						
Echocardiography performance	160 (75.1)	133 (71.1)	166 (66.4)	167 (73.6)	626 (71.4)	0.170
Severe LV dysfunction	19 (11.9)	15 (11.3)	28 (16.9)	26 (15.6)	88 (14.1)	0.410
LV hypertrophy	9 (5.6)	7 (5.3)	9 (5.4)	6 (3.6)	31 (5.0)	0.822
Mitral regurgitation	9 (5.6)	9 (6.8)	8 (4.8)	18 (10.8)	44 (7.0)	0.149
Tricuspid regurgitation	3 (1.9)	5 (3.8)	5 (3.0)	8 (4.8)	21 (3.4)	0.518
Pulmonary hypertension	5 (3.1)	12 (9)	7 (4.2)	11 (6.6)	35 (5.6)	0.126
**Results of angiography**						
Angiography performance	154 (72.3)	119 (63.6)	164 (65.6)	153 (67.4)	590 (67.3)	0.274
Measure of CAD, no or non-significant	9 (5.8)	6 (5.0)	5 (3.0)	6 (3.9)	26 (4.4)	0.618
One vessel	43 (27.9)	36 (30.3)	55 (33.5)	52 (34.0)	186 (31.5)	
Two vessels	51 (33.1)	38 (31.9)	43 (26.2)	53 (34.6)	185 (31.4)	
Three vessels/LM	51 (33.1)	39 (32.8)	61 (37.2)	42 (27.5)	193 (32.7)	
**Type of treatment**						
Noninvasive	45 (21.1)	57 (30.5)	61 (24.4)	53 (23.3)	216 (24.6)	0.164
PCI	147 (69.0)	116 (62.0)	159 (63.6)	158 (69.6)	580 (66.1)	
CABG	21 (9.9)	14 (7.5)	30 (12.0)	16 (7.0)	81 (9.2)	

The data are presented as *n* (%) unless otherwise stated. CABG—coronary artery bypass graft, CAD—coronary artery disease, CHF—chronic heart failure, CIHD—chronic ischemic heart disease, COPD—chronic obstructive pulmonary disease, GI—gastrointestinal, IHD—ischemic heart disease, LM—left main, LOS—length of stay, LV—left ventricular, PCI—percutaneous coronary intervention, PVD—peripheral vascular disease, SD—standard deviation, STEMI—ST-elevation myocardial infarction. Legend: −1 m—one-month prior to Ramadan, Ramadan—month of Ramadan, +1 m—one-month post-Ramadan, +2 m—two-months post-Ramadan.

## Data Availability

The data presented in this study are available on request from the corresponding author. The data are not publicly available due to privacy.

## Supplementary Materials

The following supporting information can be downloaded at: https://www.mdpi.com/article/10.3390/jcm11175145/s1. Table S1: Study time periods specified per year according to the Gregorian calendar. Table S2: Baseline characteristics of the non-Muslim study population by period. Table S3: Results of the multivariate models for one-month mortality: (A) for the Muslim population and (B) for the non-Muslim population.
