# Supplementary material for: The Association between Acute Myocardial Infarction-Related Outcomes and the Ramadan Period: A Retrospective Population-Based Study
Source: J Clin Med. 2022 Aug 31;11(17):5145. doi: 10.3390/jcm11175145 (PMC9457160; doi:10.3390/jcm11175145)
Supplement: Supplementary file 1 [file jcm-11-05145-s001.zip › jcm-1875293-supplementary.pdf]

Supplementary Table S1. Study time-periods specified per year according to the Gregorian calendar.

Year	Period*				Total (days)
	-1m	Ramadan	+1m	+2m	
2002	7-Oct-2002 - 5-Nov-2002	6-Nov-2002 - 5-Dec-2002	6-Dec-2002 - 4-Jan-2003	5-Jan-2003 - 3-Feb-2003	116
2003	26-Sep-2003 - 26-Oct-2003	27-Oct-2003 - 26-Nov-2003	27-Nov-2003 - 27-Dec-2003	28-Dec-2003 - 27-Jan-2004	120
2004	16-Sep-2004 - 15-Oct-2004	16-Oct-2004 - 14-Nov-2004	15-Nov-2004 - 14-Dec-2004	15-Dec-2004 - 13-Jan-2005	116
2005	5-Sep-2005 - 4-Oct-2005	5-Oct-2005 - 3-Nov-2005	4-Nov-2005 - 3-Dec-2005	4-Dec-2005 - 2-Jan-2006	116
2006	25-Aug-2006 - 23-Sep-2006	24-Sep-2006 - 23-Oct-2006	24-Oct-2006 - 22-Nov-2006	23-Nov-2006 - 22-Dec-2006	116
2007	14-Aug-2007 - 12-Sep-2007	13-Sep-2007 - 12-Oct-2007	13-Oct-2007 - 11-Nov-2007	12-Nov-2007 - 11-Dec-2007	116
2008	4-Auj-2008 - 1-Sep-2008	2-Sep-2008 - 30-Sep-2008	1-Oct-2008 - 29-Oct-2008	30-Oct-2008 - 27-Nov-2008	112
2009	23-Jul-2009 - 21-Aug-2009	22-Aug-2009 - 20-Sep-2009	21-Sep-2009 - 20-Oct-2009	21-Oct-2009 - 19-Nov-2009	116
2010	12-Jul-2010 - 10-Aug-2010	11-Aug-2010 - 9-Sep-2010	10-Sep-2010 - 9-Oct-2010	10-Oct-2010 - 8-Nov-2010	116
2011	3-Jul-2011 - 31-Jul-2011	1-Aug-2011 - 29-Aug-2011	30-Aug-2011 - 27-Sep-2011	29-Sep-2011 - 26-Oct-2011	112
2012	20-Jun-2012 - 19-Jul-2012	20-Jul-2012 - 18-Aug-2012	19-Aug-2012 - 17-Sep-2012	18-Sep-2012 - 17-Oct-2012	116
2013	9-Jun-2013 - 8-Jul-2013	9-Jul-2013 - 7-Aug-2013	8-Aug-2013 - 6-Sep-2013	7-Sep-2013 - 6-Oct-2013	116
2014	30-May-2014 - 28-Jun-2014	29-Jun-2014 - 28-Jul-2014	29-Jul-2014 - 27-Aug-2014	18-Aug-2014 - 26-Sep-2014	116
2015	20-May-2015 - 17-Jun-2015	18-Jun-2015 - 16-Jul-2015	17-Jul-2015 - 14-Aug-2015	15-Aug-2015 - 12-Sep-2015	112
2016	9-May-2016 - 6-Jun-2016	7-Jun-2016 - 5-Jul-2016	6-Jul-2016 - 3-Aug-2016	4-Aug-2016 - 1-Sep-2016	112
2017	28-Apr-2017 - 26-May-2017	27-May-2017 - 24-Jun-2017	25-Jun-2017 - 23-Jul-2017	24-Jul-2017 - 21-Aug-2017	112
**Total (days)**	**460**	**460**	**460**	**460**	**1840**

Legend: -1m – one-month prior to Ramadan, Ramadan – month of Ramadan, +1m – one-month post-Ramadan, +2m – two-months post-Ramadan.

Ramadan fast begins and ends on the evening of specified date.

Supplementary Table S2. Baseline characteristics of the Non-Muslim study population by period.

	-1 m	Ramadan	+ 1 m	+ 2 m	Total	p
N	1137	1179	1305	1333	4954	
**Demographics**						
Age, Mean (SD)	68.41 (13.38)	68.56 (13.54)	69.06 (13.56)	69.10 (13.64)	68.80 (13.53)	0.483
65-75	299 (26.3)	266 (22.6)	289 (22.1)	311 (23.3)	1165 (23.5)	0.279
≥75	386 (33.9)	422 (35.8)	494 (37.9)	491 (36.8)	1793 (36.2)	
Sex, Males	759 (66.8)	796 (67.5)	842 (64.5)	867 (65.0)	3264 (65.9)	0.351
**Cardiac diseases**						
Cardiomegaly	143 (12.6)	125 (10.6)	139 (10.7)	143 (10.7)	550 (11.1)	0.353
Supraventricular arrhythmias	211 (18.6)	244 (20.7)	234 (17.9)	248 (18.6)	937 (18.9)	0.329
CHF	218 (19.2)	241 (20.4)	313 (24.0)	312 (23.4)	1084 (21.9)	0.009
Pulmonary heart disease	130 (11.4)	126 (10.7)	148 (11.3)	159 (11.9)	563 (11.4)	0.810
CIHD	939 (82.6)	970 (82.3)	1077 (82.5)	1060 (79.5)	4046 (81.7)	0.128
**Cardiovascular risk factors**						
Renal diseases	142 (12.5)	174 (14.8)	196 (15.0)	206 (15.5)	718 (14.5)	0.169
Diabetes mellitus	480 (42.2)	513 (43.5)	616 (47.2)	618 (46.4)	2227 (45.0)	0.043
Dyslipidemia	841 (74.0)	848 (71.9)	945 (72.4)	943 (70.7)	3577 (72.2)	0.354
Hypertension	624 (54.9)	703 (59.6)	758 (58.1)	742 (55.7)	2827 (57.1)	0.072
Obesity	210 (18.5)	235 (19.9)	288 (22.1)	279 (20.9)	1012 (20.4)	0.156
Smoking	450 (39.6)	438 (37.2)	493 (37.8)	506 (38.0)	1887 (38.1)	0.666
PVD	166 (14.6)	181 (15.4)	213 (16.3)	191 (14.3)	751 (15.2)	0.494
Family history of IHD	94 (8.3)	88 (7.5)	92 (7.0)	101 (7.6)	375 (7.6)	0.726
**Other disorders**						
COPD	86 (7.6)	105 (8.9)	112 (8.6)	142 (10.7)	445 (9.0)	0.055
Neurological disorders	220 (19.3)	248 (21.0)	262 (20.1)	291 (21.8)	1021 (20.6)	0.444
Malignancy	54 (4.7)	69 (5.9)	63 (4.8)	66 (5.0)	252 (5.1)	0.587

Anemia	533 (46.9)	582 (49.4)	680 (52.1)	671 (50.3)	2466 (49.8)	0.075
GI bleeding	32 (2.8)	46 (3.9)	42 (3.2)	35 (2.6)	155 (3.1)	0.279
Schizophrenia/Psychosis	20 (1.8)	18 (1.5)	25 (1.9)	18 (1.4)	81 (1.6)	0.681
Alcohol/drug addiction	32 (2.8)	35 (3.0)	33 (2.5)	37 (2.8)	137 (2.8)	0.927
History of malignancy	79 (6.9)	84 (7.1)	74 (5.7)	94 (7.1)	331 (6.7)	0.402
**Administrative characteristics of the hospitalization**						
LOS, >7 days	540 (47.5)	585 (49.6)	630 (48.3)	620 (46.5)	2375 (47.9)	0.463
STEMI	454 (39.9)	463 (39.3)	532 (40.8)	543 (40.7)	1992 (40.2)	0.852
**Results of echocardiography**						
Echocardiography performance	808 (71.1)	848 (71.9)	909 (69.7)	918 (68.9)	3483 (70.3)	0.337
Severe LV dysfunction	110 (13.6)	112 (13.2)	145 (16.0)	117 (12.7)	484 (13.9)	0.202
LV hypertrophy	51 (6.3)	51 (6.0)	44 (4.8)	57 (6.2)	203 (5.8)	0.521
Mitral regurgitation	46 (5.7)	58 (6.8)	77 (8.5)	77 (8.4)	258 (7.4)	0.084
Tricuspid regurgitation	45 (5.6)	39 (4.6)	44 (4.8)	48 (5.2)	176 (5.1)	0.811
Pulmonary hypertension	79 (9.8)	96 (11.3)	96 (10.6)	83 (9.0)	354 (10.2)	0.424
**Results of angiography**						
Angiography performance	743 (65.3)	749 (63.5)	799 (61.2)	775 (58.1)	3066 (61.9)	0.002
Measure of CAD, No or non-significant	33 (4.4)	24 (3.2)	40 (5.0)	34 (4.4)	131 (4.3)	
One vessel	182 (24.5)	182 (24.3)	172 (21.5)	195 (25.2)	731 (23.8)	0.322
Two vessels	204 (27.5)	190 (25.4)	208 (26.0)	218 (28.1)	820 (26.7)	
Three vessels/ LM	324 (43.6)	353 (47.1)	379 (47.4)	328 (42.3)	1384 (45.1)	
**Type of treatment**						
Noninvasive	353 (31.0)	363 (30.8)	439 (33.6)	496 (37.2)	1651 (33.3)	
PCI	665 (58.5)	695 (58.9)	725 (55.6)	706 (53.0)	2791 (56.3)	0.014
CABG	119 (10.5)	121 (10.3)	141 (10.8)	131 (9.8)	512 (10.3)	

The data are presented as n (%) unless otherwise stated. CABG - coronary artery bypass graft, CAD - coronary artery disease, CHF - chronic heart failure, CIHD - chronic ischemic heart disease, COPD - chronic obstructive pulmonary disease, GI - gastrointestinal, IHD - ischemic heart disease, LM - left main, LOS - length of stay, LV - left ventricular, PCI - percutaneous coronary intervention, PVD - peripheral vascular disease SD - standard deviation, STEMI – ST-elevation myocardial infarction.

Legend: -1m – one-month prior to Ramadan, Ramadan – month of Ramadan, +1m – one-month post-Ramadan, +2m – two-months post-Ramadan.

Supplementary Table S3. Results of the multivariate models for one-month mortality: A) for the Muslim population and B) for Non-Muslim population.

A)

Parameter	B (SE)	AdjOR	(95% CI)	p
**Period**				
-1m		1 (ref.)		
Ramadan	0.804 (0.545)	2.234	(0.767 ; 6.501)	0.140
+1m	0.997 (0.504)	2.709	(1.008 ; 7.280)	0.048
+2m	0.420 (0.555)	1.522	(0.513 ; 4.514)	0.449
**Year of AMI Event**				
2002		1 (ref.)		
2003	0.858 (1.099)	2.358	(0.274 ; 20.317)	0.435
2004	0.122 (1.167)	1.130	(0.115 ; 11.134)	0.917
2005	-1.348 (1.643)	0.260	(0.010 ; 6.505)	0.412
2006	0.415 (1.358)	1.514	(0.106 ; 21.676)	0.760
2007	0.329 (1.292)	1.390	(0.110 ; 17.498)	0.799
2008	1.293 (1.136)	3.645	(0.393 ; 33.806)	0.255
2009	-0.256 (1.186)	0.774	(0.076 ; 7.905)	0.829
2010	1.305 (1.166)	3.688	(0.375 ; 36.276)	0.263
2011	-0.319 (1.342)	0.727	(0.052 ; 10.075)	0.812
2012	0.522 (1.182)	1.686	(0.166 ; 17.088)	0.659
2013	0.758 (1.212)	2.134	(0.198 ; 22.976)	0.532
2014	2.147 (1.115)	8.559	(0.962 ; 76.170)	0.054
2015	1.805 (1.207)	6.079	(0.571 ; 64.749)	0.135
2016	0.761 (1.341)	2.141	(0.155 ; 29.648)	0.570

2017	0.436 (1.134)	1.546	(0.167 ; 14.277)	0.701
**Patient characteristics**				
Age, one year increase	0.055 (0.015)	1.057	(1.027 ; 1.088)	<0.001
CIHD	-0.994 (0.402)	0.370	(0.169 ; 0.813)	0.013
Renal diseases	1.230 (0.420)	3.420	(1.502 ; 7.786)	0.003
Anemia	0.801 (0.353)	2.228	(1.115 ; 4.449)	0.023
GI bleeding	1.981 (0.626)	7.248	(2.124 ; 24.733)	0.002
Type of AMI, STEMI vs. NSTEMI	1.208 (0.337)	3.348	(1.729 ; 6.482)	<0.001
Severe LV dysfunction	1.236 (0.494)	3.441	(1.308 ; 9.056)	0.012

B)

**Parameter**	**B (SE)**	**AdjOR**	**(95% CI)**	**p**
**Period**				
-1m		1 (ref.)		
Ramadan	0.327 (0.176)	1.387	(0.982 ; 1.960)	0.064
+1m	0.283 (0.175)	1.327	(0.943 ; 1.870)	0.105
+2m	0.304 (0.172)	1.355	(0.968 ; 1.897)	0.077
**Year of AMI Event**				
2002		1 (ref.)		
2003	0.447 (0.310)	1.563	(0.851 ; 2.871)	0.150
2004	0.409 (0.308)	1.505	(0.823 ; 2.752)	0.184
2005	-0.101 (0.329)	0.904	(0.474 ; 1.723)	0.759
2006	0.196 (0.326)	1.216	(0.642 ; 2.306)	0.548
2007	-0.017 (0.357)	0.983	(0.489 ; 1.977)	0.962
2008	-0.720 (0.423)	0.487	(0.212 ; 1.115)	0.089
2009	0.402 (0.344)	1.495	(0.762 ; 2.936)	0.242

2010	-0.274 (0.355)	0.761	(0.379 ; 1.526)	0.441
2011	-0.094 (0.402)	0.910	(0.414 ; 2.001)	0.815
2012	0.095 (0.352)	1.100	(0.552 ; 2.190)	0.787
2013	0.649 (0.344)	1.914	(0.976 ; 3.753)	0.059
2014	0.872 (0.348)	2.391	(1.209 ; 4.729)	0.012
2015	0.747 (0.368)	2.110	(1.026 ; 4.340)	0.042
2016	0.214 (0.378)	1.238	(0.591 ; 2.597)	0.571
2017	0.344 (0.350)	1.411	(0.710 ; 2.804)	0.326
**Patients characteristics**				
Age, one year increase	0.038 (0.006)	1.038	(1.027 ; 1.05)	<0.001
CIHD	-0.529 (0.143)	0.589	(0.445 ; 0.78)	<0.001
Renal diseases	1.314 (0.130)	3.719	(2.882 ; 4.800)	<0.001
Dyslipidemia	-0.500 (0.122)	0.607	(0.478 ; 0.770)	<0.001
PVD	0.499 (0.141)	1.647	(1.249 ; 2.172)	<0.001
Malignancy	0.397 (0.202)	1.487	(1.001 ; 2.211)	0.049
Anemia	0.490 (0.132)	1.633	(1.261 ; 2.114)	<0.001
GI bleeding	0.528 (0.253)	1.696	(1.032 ; 2.787)	0.037
Type of AMI, STEMI vs. NSTEMI	0.890 (0.133)	2.435	(1.875 ; 3.162)	<0.001
Severe LV dysfunction	1.167 (0.184)	3.213	(2.243 ; 4.603)	<0.001
Mitral regurgitation	0.642 (0.238)	1.900	(1.193 ; 3.026)	0.007
Type of treatment, invasive vs. non-invasive	-0.471 (0.162)	0.624	(0.455 ; 0.857)	0.004

AdjOR – adjusted odds ratio, AMI – acute myocardial infarction, B – regression coefficient, CI – confidence interval, CIHD - chronic ischemic heart disease, GI - gastrointestinal, LV - left ventricular, NSTEMI – non-ST-elevation myocardial infarction, PVD - peripheral vascular disease, Ref. – reference group, SE – standard error, STEMI – ST-elevation myocardial infarction.

Legend: -1m – one-month prior to Ramadan, Ramadan – month of Ramadan, +1m – one-month post-Ramadan, +2m – two-months post-Ramadan.
